# Evaluation of effect on stability of implants with and without platelet rich fibrin using a resonance frequency analyzer - An in-vivo study

**DOI:** 10.1016/j.heliyon.2024.e27971

**Published:** 2024-03-20

**Authors:** Mounica Priya Anapu, Kaleswara Rao Atluri, Sunil Chandra Tripuraneni, Rakhi Issrani, Alzarea K. Bader, Zainab A. Alkhalaf, Mohammed Ghazi Sghaireen, Namdeo Prabhu, Raed Rbea DH Alshammari, Ghosoun Khalid, Ghada Matab, Farida Habib Khan

**Affiliations:** a Department of Prosthodontics, Drs. Sudha and Nageswara Rao Siddhartha Institute of Dental Sciences, Chinnaoutapalli, India; b Department of Preventive Dentistry, College of Dentistry, Jouf University, Sakaka, Kingdom of Saudi Arabia; c Department of Prosthetic Dental Sciences, College of Dentistry, Jouf University, Sakaka, Kingdom of Saudi Arabia; d Department of Prosthetic Dental Sciences, Ministry of Health, Kingdom of Saudi Arabia; e Department of Oral & Maxillofacial Surgery and Diagnostic Sciences, College of Dentistry, Jouf University, Sakaka, Kingdom of Saudi Arabia; f Department of Research Analytics, Saveetha Dental College and Hospitals, Saveetha Institute of Medical and Technical Sciences, Saveetha University, Chennai, India; g College of Medicine, University of Hail, Hail, Kingdom of Saudi Arabia; h Department of Family and Community Medicine, College of Medicine, University of Hail, Hail, Kingdom of Saudi Arabia

**Keywords:** Osseointegration, Stability, Platelet-derived growth factor

## Abstract

**Background:**

Although the conventional replacement for lost teeth has been partial or full dentures, the need for a fixed, esthetic, and functional restoration makes dental implants a reliable alternative.

**Aim:**

To evaluate the initial and final stability of platelet rich fibrin coated implants using resonance frequency analyzer.

**Method:**

ology: Thirteen patients with two or more missing teeth were informed about the procedure, and a consent form was obtained after cone beam computer tomography evaluation. Blood was drawn from the anticubital area of the patient, which was centrifuged to obtain platelet-rich fibrin. In all, 26 implants were placed, among which 13 were platelet-rich fibrin-coated (test group) and 13 were without platelet-rich fibrin (control group), and implant stability quotient values were recorded.

**Results:**

The mean age of the patients was 34.4 (SD = 4.28). Majority of the patients were males (9; 69.2%) whereas there were only four (30.8%) female patients. When comparison between overall primary implant stability with and without PRF was done, the mean difference was 5.12 and this difference was not statistically significant (p = 0.221) whereas a statistically significant difference (p = 0.019) was found when comparison between overall secondary implant stability was done with and without PRF. The primary and secondary stability values for the control group were 69.18 ± 7.45 and 73.84 ± 8.21 respectively, and the primary and secondary stability values for the test group were 64.06 ± 12.66 and 81.49 ± 7.61 respectively, which showed statistically significant differences among the groups. The difference in these values signify that primary stability is more in control group whereas secondary stability is more in case group. This signifies that PRF enhances the stability of implant.

**Conclusion:**

Implants coated with platelet-rich fibrin exhibited better osseointegration than implants without platelet-rich fibrin.

## 1 Introduction

Human life expectancy has increased with advancements in medical research. Greater proportions of those who live longer are partial or completely edentulous and are traditionally treated with removable prostheses or fixed dental prostheses. The drawbacks of traditional removable prostheses (retention, stability, removal and replacement daily) and fixed dental prostheses (effects on tooth structure preservation) make dental implants a viable choice [1]. Oral implants are a widely used therapeutic option for the replacement of lost or missing teeth.

A successful implant procedure requires osseointegration. The term "osseointegration" was defined as "a direct structural and functional connection between ordered, living bone and the surface of a load-carrying implant" by Branemark in 1985 [2]. Two levels of implant stability have been defined: "primary" stability and "secondary" stability [3]. Primary implant stability is recognized as a crucial criterion for successful osseointegration. An implant's primary stability is caused by mechanical engagement with the cortical bone. This ensures bone repair by preventing fibro-osseointegration between the implant and bone. Primary stability is a requirement for secondary stability. Secondary stability is biological stability that occurs through bone remodeling and regeneration during the healing phase. An additional gain in stability occurs due to bone growth surrounding the implant, which determines the time of functional loading [4]. Primary implant stability and avoidance of micromotion during early healing ensures successful osseointegration. For this purpose, implants are kept unloaded for 3–4 months in the mandible and 6–8 months in the maxilla [5,6].

The surface chemistry of the implants can be altered to increase the degree of bone-to-implant contact (BIC), which enhances the healing process. Another strategy to improve BIC is to add biomolecules and growth factors to the implant surface. These changes can be introduced to the implant surface design and topography. Bone morphogenetic proteins or cell adhesion molecules applied to the implant surface may promote osteoblast development and enhance functional integration [7]. Platelet-rich products have resulted in enhanced bone regeneration and quicker osseointegration of the implant, which improves the durability and maintenance of dental implants by boosting the BIC [8]. In implant dentistry, intense research focuses on innovative materials and technologies to improve treatment outcomes. This simultaneously reduces morbidity, biological complications, and surgical times [9].

Platelet-rich fibrin (PRF) is an autologous second-generation platelet concentrate that is composed of a polymerized fibrin matrix within a molecular structure that contains platelets, leukocytes, cytokines, and circulating stem cells that release growth factors, such as platelet-derived growth factor, vascular endothelial growth factor, transforming growth factor-β, and insulin-like growth factor, which promote soft and hard tissue healing [10]. PRF acts as a biodegradable scaffold that promotes the development of microvascularization, which can guide epithelial cell migration to its surface. The growth factors released by these platelets are chemoattractants for osteoblast precursor cells. The major cellular event in osteogenesis is stimulation of cellular attachment, proliferation, differentiation, and deposition of bone matrix, which modulate the healing process. These processes promote stem cell proliferation, which in turn encourages the appearance of capillary sprouts in endothelial cells. PRF is used as biodegradable scaffold in wound healing, resorbable membrane for guided bone regeneration, treatment of infra bony defects, seal for undetected sinus openings, biologic connect in surgical sites, biologic adhesive to hold particles together when used along with β-TCP. PRF concentrates are also been applied in dermatology, pain management, sports medicine, plastic surgery, cardiac surgery and urology [11,12].

Attaining and maintaining implant stability is essential for the success of a dental implant. Several methods by which implant stability is assessed include clinical evaluation of cutting resistance during implant insertion, reverse torque testing, the percussion test and the periotest [13,14]. Resonance frequency analysis (RFA) is a non-invasive diagnostic technology that uses vibration and structural principle analysis to determine implant stability and bone density over time. The transducer, a magnet on top of the metallic rod, is placed into an implant in the magnetic RFA device. From a wireless probe, a magnetic pulse excites the magnet, which lasts for 1 ms. The excitation vibrates the peg and then the magnet creates an electric voltage in the coil of the probe. The voltage measured by the resonance frequency analyzer is the measured signal. This approach determines the implant stability by detecting the resonance frequency of the implant-bone complex or by reading an Implant Stability Quotient (ISQ) value provided by the RFA (*Osstell ISQ, Sweden*) [15].

As of today majority of studies focuses on placement of PRF in the combination with graft material for sinus lift in the areas of pneumatization of the sinus walls [[16], [17], [18]]. Studies were obscure in determining the final stability of the implants in different aspects along with PRF placement. Thus, the purpose of this study was to determine the effect of platelet-rich fibrin on implant stability and marginal bone level using a RFA.

## 2 Materials and methods

### 2.1 Study design

A randomized prospective clinical study was used for this study. The duration of the study was 1 year (January 2022 to January 2023). Ethical approval no. IEC/DRS.S&NRSIDS/2021/PG/18 was received from the institutional review board and all the procedures in this study complied with the Helsinki Declaration.

### 2.2 Sample population and characteristics

The patients reporting to the Department of Prosthodontics for the replacement of the missing teeth with dental implants were screened. Patients having two or more teeth missing in the same arch, represented both genders and ranged in age from 25 to 55 years were selected and screened for eligibility to participate in the present study whereas patients with any lesion in the oral cavity, inadequate residual bone volume in the desired area for receiving implants, with any parafunctional habits and behavior and with any systemic diseases like hypertension, diabetes were excluded from the study.

Thirteen patients were selected who met the selection criteria. The participants were selected by non-probability convenience sampling from patients who met the selection criteria. The samples were divided into two groups, test group (patients were subjected to PRF placement in the osteotomy site during implant placement) and control group (patients were subjected to only implant placement without PRF placement).

### 2.3 Procedure

A thorough case history was compiled and evaluated. Implant placement was performed after proper diagnosis and treatment planning. To determine the patient's fitness for implant surgery, complete hemogram, INR, ESR, PCV, bleeding time, clotting time, HbA1c, HIV, and hepatitis tests were performed. Diagnostic casts, intraoral periapical radiographs, panorama, and CBCT scans were performed to assess the quality and quantity of bone at the implant site. Once implant treatment was determined to be feasible, patients were informed about the procedure, benefits, maintenance, and post-operative care. Then, informed consent was obtained from each patient.

### 2.4 PRF preparation protocol

The PRF was prepared prior to implant placement at the surgical site. PRF was created in accordance with Choukroun's specifications. Following the Choukroun et al. [15] PRF preparation criteria, 10 mL of venous blood was drawn from the anticubital vein of each of the ten individuals with a 22 gauge syringe and transferred to the test tube without anticoagulant [19]. The blood sample was immediately centrifuged for 12 min at 3000 revolutions per minute (RPM). The protocols such as equal rotor sizes, angulation of tubes and tube design were followed throughout the study. The dimensions of rotor, rotor angulation for test tube holder, RPM and time were maintained constant to gain similar relative centrifugal force (RCF) for all the groups [20].

RCF = 11.18 x r x (n/1000) ^2^

RCF depends on RPM, rotor dimensions and rotor angulation. As the radius (r) increases, the RCF also increases. Similarly with RPM (n). It is worth noting that the centrifugal speed reduces the size of clot formed with large number of cell count in it [21].

PRF membranes were produced utilizing a protocol of 3000 RPM for 12 min (RCF clot = 400 g) using Swing Out Head centrifugation device (45^o^ rotor angulation), 30 mm radius at clot, 60 mm at maximum (*REMI Centrifuge, Maharashtra, India*). Three layers were formed after centrifugation, the fibrin clot that had formed in the middle of the tube was removed, and the blood cell remnants were discarded. The PRF clot was then placed in the PRF box (*Waldent, India*) to obtain the PRF membrane.

**Fig. 1:**
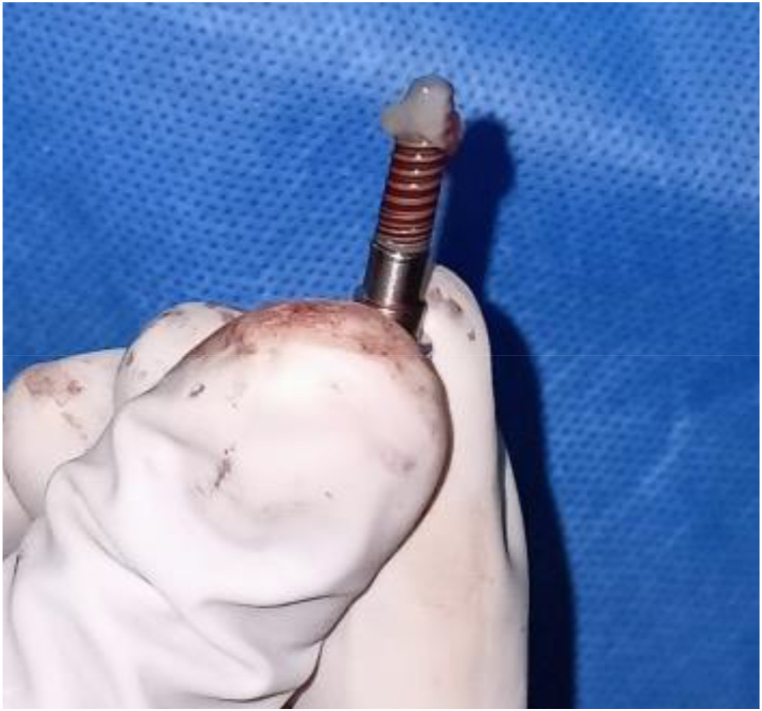
Platelet Rich Fibrin applied to implant.

Before the procedure was initiated, antisepsis was performed extra-orally with 5% povidone iodine solution and intraorally with 0.2% chlorhexidine mouth rinse. Anesthesia was induced using a 2% lidocaine hydrochloride solution with 1:80,000 adrenaline. In both groups, a full-thickness mucoperiosteal flap was reflected, and sequential osteotomy was performed to the desired length and diameter, which were preplanned using CBCT. In the test group, the PRF membrane was applied on the implant surface (*Alphabio, Isreal),* as shown in Fig. 1, later inserted into the surgical site, and the flap was approximated with a simple interrupted suture. In the control group, implants were inserted into the prepared osteotomy site, and the flaps were sutured. A minimum inter-implant distance of 3 mm was maintained.

**Fig. 2:**
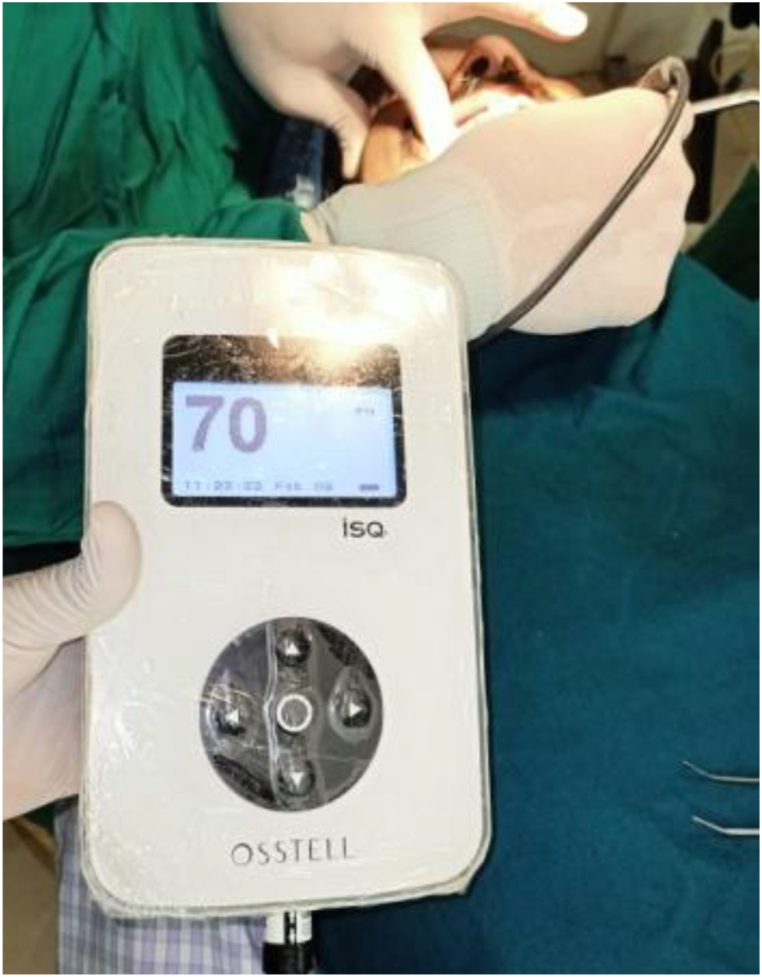
Smart peg is placed and RFA used to assess the implant stability.

RFA (*Osstell ISQ, Sweden*) was used to assess implant stability as shown in Fig. 2. The implant stability quotients were calculated using measurements obtained at the mesial, distal, buccal and lingual sites. RFA measurements were obtained immediately after implant placement and after 4 months for mandible whereas 6 months for maxilla after the surgery for both the groups. The scale was numbered from 0 to 100. The higher the ISQ values, the more stable the implant.

### 2.5 Statistical analysis

Data were analyzed using the Statistical Package for the Social Sciences version 20.0 (IBM Corp, Armonk, NY, USA). The stability values of both groups (test and control) were compared using the independent sample *t*-test, while for the comparison of primary and secondary stability values within the same group; dependent sample *t*-test was used. P-value was set at <0.05.

## 3 Results

The mean age of the patients was 34.4 (SD = 4.28). Majority of the patients were males (9; 69.2%) whereas there were only 4 (30.8%) female patients.

When comparison between overall primary implant stability with and without PRF was done, the mean difference was 5.12 and this difference was not statistically significant (p = 0.221) whereas a statistically significant difference (p = 0.019) was found when comparison between overall secondary implant stability was done with and without PRF **(**Table 1**).**

**Table 1:** Comparison between overall primary and secondary implant stability with and without PRF.

Groups	Parameter	N	Mean	SD	Std. error mean	Mean difference	t value	p-value
Overall primary values	Primary values without PRF	13	69.18	7.45	2.06	5.12	1.25	0.221
	Primary values with PRF	13	64.06	12.66	3.51			
Overall secondary values	Secondary values without PRF	13	73.64	8.21	2.27	−7.84	−2.52	0.019, S
	Secondary values with PRF	13	81.49	7.61	2.11			

PRF Platelet-rich fibrin.

N Number of participants.

SD Standard deviation.

S Statistically significant.

The primary and secondary stability values for the control group were 69.18 ± 7.45 and 73.84 ± 8.21 respectively, and the primary and secondary stability values for the test group were 64.06 ± 12.66 and 81.49 ± 7.61 respectively, which showed statistically significant differences among the groups **(**Table 2**).**

**Table 2:** Comparison between control and test groups for overall primary and secondary implant stability.

Groups	Parameter	N	Mean	SD	Std. error mean	Mean difference	t value	p-value
Control group	Overall primary values without PRF	13	69.18	7.45	2.06	−4.46	−2.21	0.046, S
	Overall secondary values without PRF	13	73.64	8.21	2.27			
Test group	Overall primary values with PRF	13	64.06	12.66	3.51	−17.43	−3.94	0.002, S
	Overall secondary values with PRF	13	81.49	7.61	2.11			

PRF Platelet-rich fibrin.

N Number of participants.

SD Standard deviation.

S Statistically significant.

**Fig. 3:**
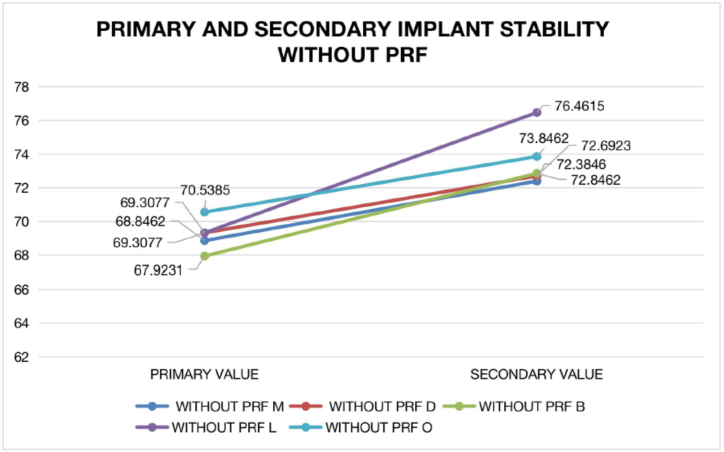
Primary and secondary implant stability without PRF.

**Fig. 4:**
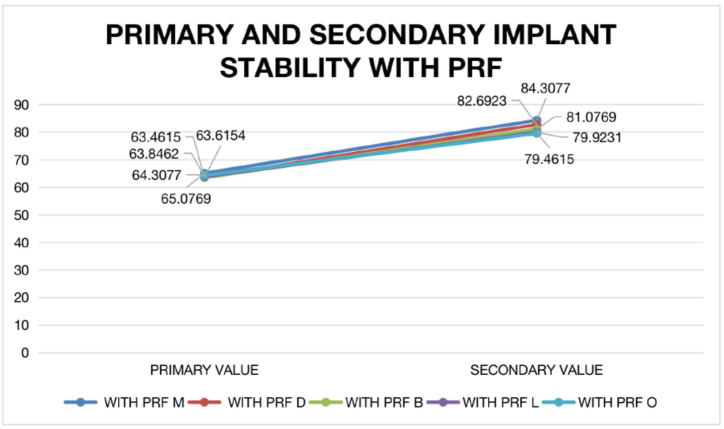
Primary and secondary implant stability with PRF.

Fig. 3and 4 shows primary and secondary implant stability without PRF and with PRF respectively.

## 4 Discussion

The purpose of this study was to induce or stimulate the regenerating capability of surrounding tissues at the bone-implant interface, which truly influences the effectiveness and lifespan of the implant, by inserting PRF membrane at the osteotomy site. By enhancing BIC, PRF membrane functions as a control release of growth factors over a period of seven days, which improves and speeds up osseous healing. Kim SG et al. [22], Zechner W et al. [23] and Lee JW et al. [24] found that platelet-rich plasma group had greater BIC than the control group in their studies on dogs and pigs, respectively. However, according to Weibrich G et al., the effect of platelet concentrate is not beneficial in accelerating the osseointegration of end-osseous dental implants as shown by their study on New Zealand white rabbits [25]. Also, in the study on beagle dogs by Streckbein P et al., no statistically significant differences was noted between the platelet-rich plasma and non-platelet-rich plasma groups on comparing the histological and histomorphometric specimens of all other implant sites in terms of peri-implant bone remodeling and the consequent bone-implant contact rates [26].

In this study, stability ratings of the 26 implants were noted immediately following implant insertion and after four to six months of implant placement. Both the test and control groups showed ISQ values greater than 60 at baseline which ensured that the implant was stable in the osteotomy site. Similar findings were noted by Oncu E et al. which concluded that PRF application increased implant stability during early healing period as evidenced by higher ISQ values [1]. Also Pirpir C et al. found that the application of concentrated growth factors seemed to accelerate osseointegration as evidenced by higher ISQ values [27].

In the present study, the primary stability ratings were essentially the same and did not exhibit any notable differences. However, the secondary stability ratings were found to increase in both groups throughout the healing period. The reason for increase in stability in this scenario is the addition of PRF which favors microvascularization, which is able to guide epithelial cell migration [1]. According to Boora P et al., PRF is regarded as a healing biomaterial with a possible favorable effect on peri-implant tissue and employed as a therapeutic adjuvant for single-tooth implant placement [28]. Also, Angelo T et al. concluded that more consistent results are obtained in the implant stability with the inclusion of PRF along with the use of other biomaterials like self-hardening calcium phosphate [29]. Interestingly, Fang J et al. reported a case wherein immediate implant placement with PRF was placed into extraction sites with periapical infection in the esthetic zone in a 34-year-old, healthy female patient with the residual root of the right maxillary central incisor. The authors recommended the use of PRF in conjunction with guided bone regeneration that can serve as a reliable and easy adjuvant for rapid implanting in diseased sockets [30]. Recent studies by Kapoor A et al. [31] and Guan S et al. [32] have concluded that PRF has a significant effect on osseointegration of dental implants during the early healing period prior to loading. Additionally, it has been found that PRF can be loaded with antibiotics to effectively release antimicrobial drugs that reduce the risk of post-operative infections. PRF loaded with antibiotics can replace or supplement systemic antibiotic therapy while still preserving the healing properties of PRF [33]. On the contrary, Anand U et al. have found a 100% overall success rate for the immediately loaded bio-activated implants placed in the mandibular posterior region and concluded that the use of platelet-rich plasma resulted in better early bone apposition around the implant as well as early osseointegration [34].

In our study, the range of mean distribution for primary stability values with PRF was found to be similar when compared to the group without PRF, indicating that there is more consistency when PRF is used. While comparing secondary stability values of implants with and without PRF, it showed mean distribution for secondary stability of implants with PRF have higher values compared to without PRF, this indicated that implants with PRF are more stable for longer. This finding is accordance with the study conducted by Hamzacebi B et al. [35], Cortese A et al. [36] and Zhou J et al. [37].

While comparing overall primary and secondary implant between the control and test groups, it is found that implants with PRF show better stability than without PRF in our study. According to the results of this study, the application of PRF leads to better osseointergration of implants when compared to the control group. The main objective of using PRF in the osteotomy site for the test group was to promote bone healing. PRF showed better soft tissue healing, which indirectly helped the wound to heal faster. Even in case of PRF membrane exposure, there is no risk of membrane infection or bone loss, as PRF ensures a second intention healing of the soft tissues. Similar results were seen in the study conducted by Alhussaini AHA wherein PRF group demonstrated significantly higher stability values than the group without PRF [14]. Also Shah SA et al. have concluded that the commercial dental implants pretreated with photo-functionalization or platelet-rich plasma exhibits a statistically significant difference in implant stability [38]. However, Monov G et al. concluded that the instillation of platelet-rich plasma during implant placement in the mandibular anterior region did not add any additional benefits [39]. Similarly, Khairy NM et al. in their study concluded that platelet-rich plasma did not significantly improve bone quality at 3 months post grafting; however platelet-rich plasma enriched bone graft were associated with superior bone density at 6 months post grafting [40]. In a recently study by Attia S et al., wherein the authors aimed to investigate the long term influence of platelet-rich plasma on dental implants after maxillary augmentation and they found no significant difference between the two groups (with platelet-rich plasma versus non-platelet-rich plasma) [41].

In the present study, an investigation on stability of implants after the PRF placement was done during the initial placement of implant and 4–6 months after implantation. The results and the time frame for adequate stability of the implant are in accordance with the study conducted by Siqueira RAC et al. which concluded that the reduction in stability is more pronounced in the first month where as it reached peak stability at 8 months [42]. This is due to biologic response of bone to surgical trauma which initially reacts by osteoclastic activity leading to decreased ISQ values followed by osteoblastic activity involving bone remodeling [2].

There are recent concepts in this study like horizontal centrifugation, which has advantages over angular centrifugation such as increase stability of gel layer, decreased surface area, cuts down waiting time, allows blood to separate more efficiently and has better gel seal in less time. Modification in the test tube led to more recent advances like Titanium Platelet Rich Fibrin, Advanced Platelet Rich Fibrin, Leukocyte Platelet Rich Fibrin, Concentrated Platelet Rich Fibrin and injectable Platelet Rich Fibrin, have more concentration of cells, reduce recovery times, but it also decreases pain and swelling and improves the overall patient experience. These are the potential areas where additional studies are needed to be built on this work [43].

### 4.1 Limitations

This study had few limitations. Firstly, sample size was small and the follow-up period was short term. Further long-term studies with larger sample should be carried out to assess the beneficial aspects of PRF when used along with implants. Secondly, region specification was not followed which would help in specifying the effect on that particular region. Finally, standardized implant sizes would have led to more accurate conclusion over the effect of PRF.

## 5 Conclusion

PRF is an autologous graft obtained from the patient's own plasma, which results in fewer rejections and thus enhances the secondary stability of implants. The bioactive proteins in platelet concentrates regulate wound healing by encouraging collagen production in the wound. PRF has consistently increased the long-term life expectancy of the implant, which provides assurance to both the dentist and the patient. An increased implant stability quotient has been observed with an adequate duration of the healing period. Increase of BIC is noticed by the difference of stability values obtained from both the groups that implies that PRF contains growth factors that enhanced the bone proliferation around the implant and soft tissue healing.

## Funding

The authors received no external/internal funding.

## Data availability statement

Data will be made available on request.

## Ethical statement

Ethical approval no. IEC/DRS.S&NRSIDS/2021/PG/18 was received from the institutional review board.

## CRediT authorship contribution statement

**Mounica Priya Anapu:** Conceptualization, Methodology, Formal analysis, Software, Investigation, Resources, Data curation, Writing – original draft. **Kaleswara Rao Atluri:** Conceptualization, Methodology, Validation, Resources, Data curation, Project administration. **Sunil Chandra Tripuraneni:** Validation, Formal analysis, Resources, Data curation, Project administration. **Rakhi Issrani:** Investigation, Visualization. **Alzarea K Bader:** Writing – review & editing. **Zainab A Alkhalaf:** Writing – review & editing. **Mohammed Ghazi Sghaireen:** Writing – review & editing. **Namdeo Prabhu:** Supervision, Project administration. **Raed Rbea DH Alshammari:** Formal analysis. **Ghosoun Khalid:** Formal analysis. **Ghada Matab:** Formal analysis. **Farida Habib Khan:** Formal analysis. All authors have read and agreed to the published version of the manuscript.

## Declaration of competing interest

The authors declare that they have no known competing financial interests or personal relationships that could have appeared to influence the work reported in this paper.

## Summary of Abbreviations

SD: Standard Deviation
PRF: Platelet Rich Fibrin
BIC: Bone to Implant Contact
RFA: Resonance Frequency Analyser
ISQ: Implant Stability Quotient
RCF: Relative Centrifugal Force
r: Radius
RPM: Revolutions per Minute
INR: International Normalized Ratio
ESR: Erythrocyte Sedimentation Rate
PCV: Packed Cell Volume
CBCT: Cone Beam Computed Tomography
β-TCP: β Tricalcium Phosphate
